# Cigarette smoking and smoking cessation in relation to risk of rheumatoid arthritis in women

**DOI:** 10.1186/ar4218

**Published:** 2013-04-22

**Authors:** Daniela Di Giuseppe, Nicola Orsini, Lars Alfredsson, Johan Askling, Alicja Wolk

**Affiliations:** 1 Division of Nutritional Epidemiology, Institute of Environmental Medicine, Karolinska Institutet, Nobels vag 13, Stockholm, 171 77, Sweden; 2 Division of Cardiovascular Epidemiology, Institute of Environmental Medicine, Karolinska Institutet, Nobels vag 13, Stockholm, 171 77, Sweden; 3 Clinical Epidemiology Unit, Department of Medicine, Rheumatology Unit d2:01, Karolinska University Hospital, Solna, 171 76, Sweden

## Abstract

**Introduction:**

Whereas the overall association between smoking and rheumatoid arthritis (RA) must be regarded as established, considerably less is known about how much smoking is needed to increase the risk of RA, that is, the effect of smoking intensity, duration and cessation.

**Methods:**

The Swedish Mammography Cohort, including 34,101 women aged 54 to 89 years, was followed up from January 1, 2003 through December 31, 2010 (219 RA cases identified). Relative risks (RR) and their 95% confidence intervals (CI) were estimated as rate ratios using Cox proportional hazards model.

**Results:**

There was a statistically significant association between smoking intensity (RR comparing 1 to 7 cigarettes/day vs never smoking 2.31 (95% CI: 1.59, 3.36)) as well as duration of smoking (comparing 1 to 25 years vs never smoking RR = 1.60 (95% CI: 1.07, 2.38)) and risk of RA. Compared to never smokers, the risk was still significantly elevated 15 years after smoking cessation (RR = 1.99 (95% CI: 1.23, 3.20)). However, among former smokers, the risk of RA seemed to be decreasing over time since stopping smoking: women who stopped smoking 15 years before the start of the follow-up had 30% lower risk of RA compared to those who stopped only a year before start of the follow-up (RR = 0.70 (95% CI: 0.24,2.02)).

**Conclusions:**

This prospective study highlights that even light cigarette smoking is associated with increased risk of RA in women and that smoking cessation may reduce, though not remove, this risk.

## Introduction

A large number of case-control studies [1-15] and fewer cohort studies [16-21] have shown that cigarette smoking is directly associated with the risk of developing rheumatoid arthritis (RA). Previous studies have mainly focused on establishing this association and assessed smoking status (current, former, and never smoker) [1,4,7,9,10,14-20] and pack-years of smoking [2,3,5,6,11-13,18,19], while less attention was given specifically to intensity [6,8,16,18,20,21] and/or duration of smoking [6,18,21]. Results on these aspects of cigarette smoking have so far indicated that the risk of developing RA increases in a dose-response manner with the intensity and the duration of smoking. From a public health risk assessment perspective, however, it is also important to understand how much smoking is needed to increase the risk. Indeed, experimental data suggest that even a small amount of smoke exposure may trigger an immune reaction leading to development of RA [22,23] and therefore, even light smoking is theoretically enough to induce RA.

Even less is known about the effect of smoking cessation on future risk of RA. Indeed, only one case-control study [6] and two prospective cohort studies [18,19] have evaluated the influence of smoking cessation on the risk of developing RA. Those results showed a decrease in risk 10 to 20 years after smoking cessation.

The aims of this study were therefore to further investigate if even light smoking is associated with increased risk of RA, and whether and to what extent smoking cessation may reduce the elevated risk of RA development among smokers. To this end, we analyzed the association between smoking (intensity, duration, pack-years) and smoking cessation and risk of RA in a prospective population-based cohort study of 34,101 women aged 54 to 89 years.

## Materials and methods

### Study population

The Swedish Mammography Cohort is a prospective cohort study established between 1987 and 1990. All women born between 1914 and 1948 and residing in two Swedish counties (Uppsala and Västmanland) received a questionnaire on diet and lifestyle. The response rate was 74% (*n *= 66,651). In 1997 a second questionnaire was sent to women still alive with additional questions on smoking history, physical activity, use of some medications, and dietary supplements. The response rate was 70%. Of the 39,227 questionnaires received, 243 (0.6%) were excluded due to incorrect or missing personal identification numbers. For the purpose of the present study, we excluded women with missing values on smoking status (*n *= 797) and women with non-RA joint conditions (*n *= 2,052, ICD-10 codes M07 to M12, M14, M45, M46, M30 to M36). We also excluded women with a diagnosis of RA (*n *= 432, ICD-10 codes M05 and M06) or who died (1,602 women identified through the Swedish Death Register) before the start of follow-up on 1 January 2003.

The final study cohort included 34,101 women aged 54 to 89 years at the start of follow-up on 1 January 2003. This study was approved by the Regional Research Ethics Board at Karolinska Institutet, and all participants gave their informed consent.

### Assessment of smoking and covariates

We used data from the 1997 questionnaire as the baseline, since smoking information was not collected in the 1987 questionnaire. Women were asked about smoking status, average number of cigarettes smoked per day at different ages (15 to 20, 21 to 30, 31 to 40, 41 to 50, and 51 to 60 years of age, and in the present year), age of starting smoking, and years since stopping smoking. Based on the data collected, we calculated the average number of cigarettes smoked per day during the smoking period (intensity) and the years of the period of smoking (duration), for both former and current smokers. Lifetime exposure to smoking was estimated by multiplying the average number of cigarettes smoked per day by the number of years the person had smoked divided by 20 (pack-years). For smoking cessation, the number of years since quitting smoking and the age at smoking cessation were calculated.

Intensity and duration were categorized according to tertiles of the distributions, while pack-years were categorized according to quartiles. Body mass index (BMI) was defined as weight in kilograms divided by the square of height in meters (kg/m^2^).

### Identification of RA cases and follow-up of the cohort

Cases of RA were identified by linking the cohort to three Swedish registers, using the unique Swedish personal identification number. The Outpatient Register (OPR) of the Swedish National Board of Health and Welfare (NBHW) started in 2001 to collect information on outpatient visits including both public and private care-givers. The Swedish Rheumatology Register (SRR) is a web-based national surveillance system started in the mid-nineties as part of standard health care. The Inpatient Register (IPR) of the NBHW collects information on hospitalizations with national coverage from 1987. Data from IPR was used only to identify prevalent cases at the start of follow-up. Based on chart reviews, approximately 90% of the register-identified cases with RA fulfilled the American College of Rheumatology criteria [24,25].

We analyzed the distribution of the annual number of newly diagnosed RA patients in the OPR (Additional file 1) and we observed the presence of a mixture of prevalent and incident cases during the first years of the OPR. We therefore decided to introduce a 2-year lag period and delay the start of the follow-up to 1 January 2003. The follow-up period ended on 31 December 2010. In sensitivity analyses, alternative lag periods (3- and 5-year) were employed, and newly diagnosed RA cases identified during the follow-up period through the IPR were included (Additional file 1).

### Statistical analysis

We used the Cox proportional hazard model to estimate the association between cigarette smoking and RA in terms of relative risk (RR) as the hazard rate ratio and its 95% CI. All RRs had age as the time scale [26,27]. Additional adjustment was done for potential RA risk factors such as menopausal status (pre- or postmenopausal), parity (0, 1, 2, or ≥ 3 children), use of alcohol (never, former, current < 2 drinks per week, or ≥ 2 drinks per week), educational level (lower than high school, high school, or university), and BMI (quartiles). To disentangle an independent effect of both years since quitting smoking and age at quitting smoking from the lifelong accumulated amount of smoking, the RR estimates were additionally adjusted for pack-years. Tests for trend were performed using the median value of each category to create a single continuous variable. We checked the proportionality of hazard over time using Schoenfeld's residuals [28]. There was no evidence of departure from the proportionality assumption. The dose-response association between years since quitting smoking and RA was further evaluated using the restricted cubic spline analysis [29].

A series of sensitivity analyses were performed to evaluate the consistency of the obtained results. First, we further delayed the start of the follow-up and we considered the inclusion as incident cases of newly diagnosed RA patients identified using hospitalization data from the IPR (Additional file 1, part A). Second, we performed a probabilistic sensitivity analysis, assuming that among newly diagnosed RA patients there are 0 to 20% prevalent RA cases. We made two assumptions regarding the smoking behavior of prevalent cases: no change in behavior, and smoking cessation (Additional file 1, part B).

We used SAS (version 9.2) to perform all the analyses, except for the restricted cubic spline analysis, which was performed in Stata (version 11.1). *P*-values lower than 0.05 were considered statistically significant.

## Results

According to the main case definition 219 cases of RA were identified during the 8-year period of follow-up (254,996 person-years). Of these, 79 (36%) were never smokers, 60 (27%) were former smokers and the remaining 80 (37%) cases were current smokers (Table 1). Former and current smokers were generally younger and were drinking more alcohol compared to never smokers. Intensity of smoking was similar among current and former smokers, while the duration of smoking was almost double among current smokers (mean 39 ± SD 8.6 vs 22 ± 12 years among former smokers).

**Table 1:** Baseline characteristics by smoking status of 34,101 women from the Swedish Mammography Cohort, 1997

	Never smokers *n *= 18,424 (54%)	Former smokers *n *= 7,817 (23%)	Current smokers *n *= 7,806 (23%)
RA cases, number	79	60	80
Age, years, mean (SD)	63.6 (9.2)	58.9 (8.2)	58.9 (8.2)
Body mass index, Kg/m^2^, mean (SD)	25.2 (3.8)	25.2 (4)	24.4 (3.9)
Age of starting smoking, years, mean (SD)		19.5 (5.8)	20.2 (6.9)
Age at stopping smoking, years, mean (SD)		41.4 (12.5)	
Postmenopausal, %	83.9	72.3	77
Post-secondary education, %	17.3	24.8	17.2
Nulliparous, %	10.2	8.6	8.5
Alcohol users, %	76.2	92.5	91.2

RA, rheumatoid arthritis.

### Association between smoking and RA: intensity, duration, and pack-years

The age-adjusted RR of developing RA was 2.24 (95% CI 1.63, 3.07) among current smokers compared with never smokers, and was little influenced by further adjustment for menopausal status, parity, alcohol use, educational level, and BMI (multivariable adjusted RR = 2.20 (95% CI 1.58, 3.04)) (Table 2). Among former smokers the multivariable adjusted risk of developing RA was 68% higher (95% CI 19%, 138%) compared to never smokers.

**Table 2:** Relative risk of rheumatoid arthritis by smoking status, intensity, duration and pack-years of cigarette smoking among (ever smokers) in the Swedish Mammography Cohort, 2003 to 2010

	Person-years^1^	Cases, number^1^	Age-adjusted relative risk	95% CI	**Multivariable relative risk**^3^	95% CI
**Smoking status**						
Never	137,162	79	1.00 (ref)		1.00 (ref)	
Former	59,466	60	1.65	1.17, 2.32	1.68	1.19, 2.38
Current	58,368	80	2.24	1.63, 3.07	2.20	1.58, 3.04
**Intensity, cigarettes/day^2^**						
Never	137,162	79	1.00 (ref)		1.00 (ref)	
1 to 7	34,032	47	2.23	1.54, 3.21	2.31	1.59, 3.36
8 to 14	32,010	45	2.23	1.54, 3.24	2.19	1.50, 3.21
> 14 (median 18)	35,586	33	1.45	0.96, 2.20	1.46	0.96, 2.23
**Duration, years**						
Never	137,162	79	1.00 (ref)		1.00 (ref)	
1 to 25	39,634	39	1.55	1.05, 2.30	1.60	1.07, 2.38
25 to 40	53,219	68	2.01	1.43, 2.83	1.99	1.40, 2.82
> 40 (median 45)	23,528	32	2.36	1.55, 3.58	2.33	1.52, 3.57
**Pack-years**						
Never	137,162	79	1.00 (ref)		1.00 (ref)	
1 to 5	27,534	28	1.67	1.08, 2.58	1.72	1.11, 2.67
6 to 13	30,613	40	2.13	1.45, 3.13	2.19	1.48, 3.25
14 to 22	36,990	35	2.10	1.40, 3.14	2.04	1.35, 3.09
> 22 (median 28)	27,628	31	1.81	1.19, 2.76	1.82	1.19, 2.79

^1^Numbers do not add up to the totals because of missing values; ^2^lifetime average among ever smokers; ^3^Adjusted for age (continuous), menopause status (pre- or postmenopausal), parity (0, 1, 2, or ≥ 3 children), alcohol use (none, former, or current), educational level (lower than high school, high school, or university), and body mass index (quartiles). Intensity, duration and pack-years were not mutually adjusted [35]. Ref, reference group.

Focusing on the intensity of cigarette smoking, measured as the lifetime average number of cigarettes smoked per day, we estimated that the risk of RA was more than twice the risk of never smokers, in both age- and multivariable-adjusted models, even for women with low intensity of smoking, that is, those who smoked 1 to 7 cigarettes per day. Regarding the duration of cigarette smoking evaluated among ever smokers, the multivariable risk for those who smoked for longer than 25 years was twice that of never smokers. The risk of developing RA among women who had smoked 1 to 5 pack-years was already 70% higher than the risk for never smokers.

We then analyzed the combined effect of intensity and duration of cigarette smoking (Table 3). Duration of smoking seemed to be a more important risk factor than smoking intensity. Light smokers (1 to 10 cigarettes per day) who smoked for many years (> 30 years) had a substantially elevated risk (RR = 2.54, 95% CI 1.76, 3.69).

**Table 3:** Multivariable adjusted relative risk and 95% confidence interval for rheumatoid arthritis by intensity and duration of cigarette smoking in the Swedish Mammography Cohort, 2003 to 2008

			Intensity			
	Cases, number	Never smokers	Cases, number	1 to 10 Cigarettes/day	Cases, number	> 10 Cigarettes/day
**Duration**						
Never smokers	79	1.00 (ref)		-		**-**
1 to 30 years (median 17)		-	38	1.96 (1.31, 2.93)	11	1.12 (0.59, 2.14)
> 30 years (median 38)		-	51	2.54 (1.76, 3.69)	25	1.56 (0.98, 2.50)

Results are presented as relative risk (95% CI) adjusted for age (continuous), menopausal status (pre-or postmenopausal), parity (0, 1, 2, or ≥ 3 children), alcohol use (none, former, or current), educational level (lower than high school, high school, or university), and body mass index (quartiles). Ref, reference group.

### Relative risk of RA following smoking cessation

**Figure 1:**
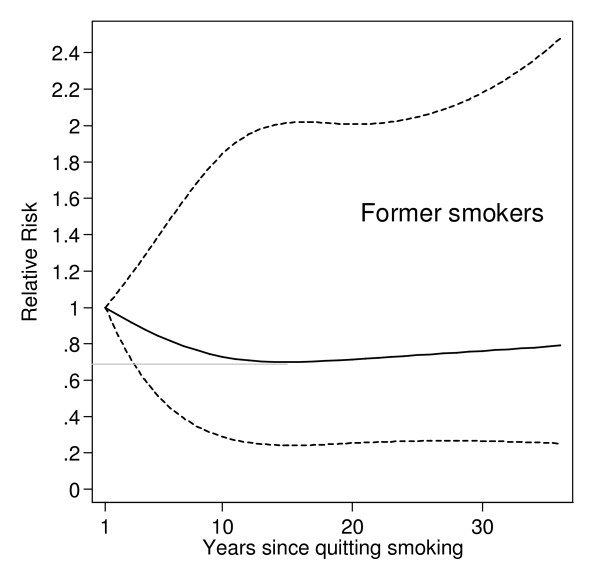
**Relative risk estimation with 95% confidence interval for the association between rheumatoid arthritis (RA) and years since stopping smoking among former smokers**. Model was adjusted for age (continuous), menopausal status (pre- or postmenopausaul), parity (0, 1, 2, or ≥ 3 children), alcohol use (none, former, or current), educational level (lower than high school, high school, or university), BMI (quartiles) and pack-years of smoking (continuous among former smokers).

The effect of smoking cessation on the risk of RA was evaluated among former smokers, with attention to the number of years since quitting smoking and to the age at smoking cessation. There was a suggestion that the risk of RA was decreasing with years since quitting smoking, although the decrease was not statistically significant (Figure 1). Those who stopped smoking 15 years before baseline in 1997 had 30% lower risk of RA compared to those who stopped only a year before, but the result was not statistically significant (RR = 0.70, 95% CI 0.24, 2.02).

However, compared to never smokers, the risk among those who stopped smoking more than 15 years before the start of follow-up was statistically significantly higher (RR = 1.99, 95% CI 1.23, 3.20) (Table 4). The risk of RA remained significantly elevated among those who stopped smoking at an early age: among those who stopped before the age of 41 years the risk was 2-fold the risk of never smokers.

**Table 4:** Relative risk of rheumatoid arthritis among former smokers by years since quitting and age at quitting cigarette smoking in the Swedish Mammography Cohort, 2003 to 2010

	Person-years^1^	Cases, number^1^	Age-adjusted relative risk	95% CI	**Multivariable relative risk**^2^	95% CI
**Never smokers**	137,162	79	1.00 (ref)		1.00 (ref)	
**Years since quitting**						
1-14	23,297	20	1.45	0.88, 2.38	1.50	0.68, 3.30
≥ 15 (median 24)	35,613	39	1.85	1.25, 2.73	1.99	1.23, 3.20
**Age at quitting**						
≤ 41 (median 32)	31,164	31	1.68	1.09, 2.59	1.94	1.18, 3.18
> 41 (median 50)	27,747	28	1.70	1.11, 2.62	2.02	1.01, 4.07

^1 ^Numbers do not add up to the totals because of missing values. ^2^Adjusted for age (continuous), menopausal status (pre- or postmenopausal), parity (0, 1, 2, or ≥ 3 children), alcohol use(none, former, or current), educational level (lower than high school, high school, or university), body mass index (quartiles), and pack-years of smoking (continuous among former smokers). Ref, reference group.

### Sensitivity analysis

Results from the sensitivity analyses performed using four additional RA case definitions, as well as results from the probabilistic sensitivity analysis, were concordant with results from the main analysis (Additional file 1, Table A and B).

## Discussion

In this population-based prospective study we observed an increased risk of RA for low levels of intensity, duration and pack-years of cigarette smoking. There was a suggestion that stopping smoking reduced the risk of RA over time among former smokers. However, compared to never smokers, the risk was still significantly elevated even among those who stopped smoking more than 15 years before the start of follow-up, and also among those who stopped smoking before age 41 years.

Our results are in agreement with a meta-analysis of smoking status [30], but some previous prospective studies found no statistically significant association between smoking and RA among current [16,20] and former [16,19,20] female smokers.

Previous studies have shown a dose response relationship between RA and smoking intensity [6,18,21], duration [6,18,21] and pack-years [2,6,9,11,14], and with low levels of smoking, which were not statistically significantly associated with RA. Our results, showing a statistically significant increased risk even for light smokers, are instead in agreement with a previous case-control study of pack-years [5] and results from a retrospective study on duration of smoking [21]. Moreover, our finding that smoking duration has a stronger impact on RA development compared to smoking intensity was in accordance with the only previous study that evaluated those aspects of smoking in the same analysis [21].

Smoking cessation has been investigated in two prospective cohorts [18,19] and one case-control study [6]. Our results, showing a statistically significantly increased risk even more than 15 years after smoking cessation, are not in agreement with these previous studies. Criswell *et al. *[19] and Stolt *et al. *[6] reported that the risk remained significantly elevated only for those who stopped smoking less than 10 years before baseline, but those studies did not take into consideration the possible confounding due to the pack-years of smoking. Costenbader *et al. *[18] showed that the risk was not statistically significant only among those who stopped smoking more than 20 years before baseline. Those results were adjusted for smoking intensity, while in the present study we preferred to adjust for pack-years, in order to take into account both duration and intensity of smoking. None of the previous studies examined the association between the age at stopping smoking and risk of RA.

The biological role of smoking in the development of RA is still unclear. However, it is known that smoking has many effects on the immune system [31,32]. In particular, smoking is a trigger for an immune response against citrullinated self-proteins, especially in the presence of *HLA-DR *shared epitope genes [22,23], which in turn might cause arthritis [4,5,33]. Our results support this biological explanation since even a relatively low amount of total smoking (1 to 5 pack-years) was associated with an almost double risk of RA. Moreover, the triggering mechanism is also in line with our results on the lack of decrease in risk after smoking cessation compared to never smoking.

The strength of this study is its prospective population-based design in which the ascertainment of exposure was independent of the ascertainment of outcome. Self-reported smoking status was validated in previous papers against cotinine-plasma levels and found to be accurate [34].

To identify newly diagnosed cases of RA during the follow-up period we used two Swedish registers, while some previous studies [16,18,19,21] used self-reported and subsequently validated RA diagnosis. However, the use of registers requires some attention, since the date of first diagnosis in the register does not always correspond to the onset of disease. The SRR, which has more accurate information on the date of onset of the disease, did not have complete coverage in the study area, and therefore it was not possible to use it as the only source of information on new RA cases. Additionally, data from the OPR was only available from 2001. We decided to start the follow-up period two years later in 2003, since we noticed an excess in the number of newly diagnosed RA patients in these years (Additional file 1, Figure App-1). This phenomenon can be explained by the presence of patients who appeared for the first time in the register but had already a previous diagnosis of RA. Another possible source of data for newly diagnosed RA cases is the IPR. However, we considered newly diagnosed RA cases identified through hospitalization as prevalent cases, and therefore we excluded them from the cohort, since RA is a disease that usually does not lead to hospitalization in the first stages. Since this assumption could be considered too strict, we considered the inclusion of these cases in sensitivity analyses (Additional file 1, part A). Results of the sensitivity analyses were consistent with observed results in the main analysis.

The gap between data collection and the start of follow-up could be considered a strength of this study. Since the onset of RA cannot usually be considered to concurring with the diagnosis date, there is the possibility that people with early symptoms of RA change their habits, leading to differential misclassification. This could explain why the increased risk among women in the higher category of intensity of smoking was only borderline statistically significant. However, an early assessment of exposure and covariates in our study 6 years before the start of follow-up should help prevent this misclassification.

Among the limitations of this study, is the fact that we only have data on women aged 54 to 89 years, and so we were unable to assess the risk of RA among younger women and men. Moreover, the number of individuals who quitted smoking was fairly limited, leading to problems of power in the estimates. Information about smoking habits was collected only to a single occasion (1997), whereas repeated measures during the study period could have been more appropriate to avoid possible non-differential misclassification of the exposure. Stratification by presence of rheumatoid factor was not possible in this study due to lack of complete information from the registers and limited statistical power. Furthermore, we had not information about shared-epitope genes. It would have been of great interest to perform such stratified analyses since evidence is growing of a greater risk of RA related to cigarette smoking when in interaction with *HLA-DR *shared-epitope genes [4,5,11].

## Conclusions

In conclusion, our study indicated that even light smoking is associated with increased risk of RA for women. In extension to this, we showed that the risk of RA was decreasing over time after smoking cessation, but compared to never smokers the risk was still statistically significantly higher. The clearly increased risk of RA development even among former smokers is another reason to persuade women not to start smoking.

## Abbreviations

BMI: body mass index; IPR: Inpatient Register; OPR: Outpatient Register; RA: rheumatoid arthritis; RR: relative risk; SRR: Swedish Rheumatology Register.

## Competing interests

The authors declare that they have no competing interests.

## Authors' contributions

DDG, NO, LA, JA, and AW participated in the study design and in writing the manuscript. DDG, NO and AW participated in the data collection. DDG analyzed the data and wrote the manuscript under the supervision of AW. DDG, NO, LA, MB, JA, and AW interpreted the data and critically reviewed the paper. All authors read and approved the final manuscript.

## Supplementary Material

Additional file 1 **Sensitivity analyses**. A series of sensitivity analyses were performed to evaluate the consistency of the reported results. In part A, different case definitions were considered. In part B, a probabilistic sensitivity analysis was performed by excluding possible prevalent cases included in the study as incident cases. Click here for file
