# Supplementary material for: Cigarette smoking and smoking cessation in relation to risk of rheumatoid arthritis in women
Source: Arthritis Res Ther. 2013 Apr 22;15(2):R56. doi: 10.1186/ar4218 (PMC3672738; doi:10.1186/ar4218)
Supplement: Additional file 1 — Sensitivity analyses. A series of sensitivity analyses were performed to evaluate the consistency of the reported results. In part A, different case definitions were considered. In part B, a probabilistic sensitivity analysis was performed by excluding possible prevalent cases included in the study as incident cases. [file ar4218-S1.DOCX]

**Appendix**

1. **Alternative outcome definitions**

Start of follow-up

The period of follow-up for this study started January 1, 2003 and ended December 31, 2010. The delay on the start of the follow-up respect to the date of the questionnaire was due to the late start of the Outpatient Register in 2001. We additionally considered a 2-year period of wash-out to avoid the inclusion of prevalent RA cases. This decision was based on the distribution of new cases per year in the Outpatient Register (Fig App-1, see below)

**Figure App-1: Frequency of newly diagnosed RA cases identified through the Outpatient Register between 2001 and 2010.**

However, we considered longer wash-out periods in sensitivity analyses.

**Figure App-2: Timeline to illustrate starts of follow-up according to the main analysis and to the different sensitivity analyses performed.**

End of follow-up

Start of follow-up in main analysis

Questionnaire

Start of

Outpatient Register

2006

2003

2001

1997

2004

2010

2-year wash-out period (main analysis)

3-year wash-out period

5-year wash-out period

Results on number of pack-years were reported in Table A for 3-year and 5-year wash-out periods: estimates for 3-year wash-out were higher than estimates in main analysis, while were lower for the 5-year period due probably to the limited number of cases. Results on intensity, duration and cessation were concordant.

Use of Inpatient Register

Data on hospitalizations from the Inpatient Register was used in the study only to identify prevalent RA cases at start of follow-up. In two sensitivity analyses (Table A, c and d) we also included newly diagnosed RA patients identified through the Inpatient Register. Results for the follow-up periods 2003-2010 and 2004-2010 were concordant with main analysis.

**TABLE A. Multivariable adjusted relative risk**‡ **and 95 percent confidence interval (in parenthesis) for four additional alternative case definitions (different time of follow-up and exclusion/inclusion of the Inpatient Register) by pack-years in the Swedish Mammography Cohort.**

|  |  |  |  | **Sensitivity a** |  | **Sensitivity b** |  | **Sensitivity c** |  | **Sensitivity d** |
| --- | --- | --- | --- | --- | --- | --- | --- | --- | --- | --- |
|  | **Cases** | **Main**  **Analysis**  **2-years w-o*** | **Cases** | **Follow-up**  **2004-2010**  **3-year w-o*** | **Cases** | **Follow-up**  **2006-2010**  **5-year w-o*** | **Cases** | **Follow-up**  **2003-2010**  **+**  **Inpatient Reg.** | **Cases** | **Follow-up**  **2004-2010**  **+**  **Inpatient Reg.** |
| **Number of cases*** | **219** |  | **172** |  | **108** |  | **299** |  | **244** |  |
| **Pack-years** |  |  |  |  |  |  |  |  |  |  |
| **Never** | 79 | 1.00(ref) | 57 |  | 44 |  | 123 |  | 95 |  |
| **1-5** | 28 | 1.72  (1.11-2.67) | 23 | 1.98  (1.21-3.25) | 11 | 1.23  (0.63-2.41) | 39 | 1.72  (1.19-2.49) | 32 | 1.88  (1.25-2.84) |
| **6-13** | 40 | 2.19  (1.48-3.25) | 35 | 2.73  (1.77-4.21) | 21 | 2.14  (1.25-3.66) | 49 | 1.95  (1.39-2.75) | 44 | 2.35  (1.62-3.40) |
| **14-22** | 35 | 2.04  (1.35-3.09) | 27 | 2.26  (1.40-3.64) | 16 | 1.73  (0.95-3.16) | 41 | 1.81  (1.25-2.62) | 33 | 1.98  (1.31-3.00) |
| **>22**  **(median 28)** | 31 | 1.81  (1.19-2.79) | 25 | 2.14  (1.32-3.48) | 13 | 1.47  (0.78-2.77) | 38 | 1.66  (1.14-2.42) | 32 | 1.93  (1.27-2.91) |

*Number of cases do not sum to the total cause of missing values for the variable pack-years.

‡ Adjusted for age (continuous) , menopause status (pre-/post-menopause), parity (0, 1, 2, ≥3 children), alcohol use(none, former, current), educational level (less than high school, high school, university), and BMI (quartiles).

*w-o = wash-out period

1. **Exclusion of potential prevalent cases**

Despite our attention on avoiding the inclusion in the analysis of prevalent cases, is still possible that some newly diagnosed RA patients identified during the follow-up period have developed the disease before the start of follow-up. To evaluate the possible changes in the estimates due to the presence of prevalent cases, we performed a probabilistic sensitivity analysis. We assumed an a priori distribution that captures our uncertainty about the amount of prevalent cases in the cohort, in the form of a uniform with values 0 to 20. We then performed simulations with 200 draws from the uniform distribution: each draw corresponded to the percentage of prevalent cases that should be randomly excluded. We then calculated the corresponding RR for pack-years. We obtained the distributions of the RR estimates according to two assumptions (median and standard deviation are reported in table B):

1. Prevalent cases did not change their smoking status (cases were excluded randomly from all categories)
2. All prevalent cases quitted smoking (cases were excluded only from the lower category)

Results from the sensitivity analyses did not differ from the main analysis.

**TABLE B.** **Median values‡ and standard deviation of the distributions of RA relative risks by pack-years according to two alternative assumptions about the behaviour of prevalent cases among incident cases (1. Prevalent cases did not changed smoking status; 2. Prevalent cases stopped smoking).**

| **Pack-years** | **Assumption 1** | **Assumption 2** |
| --- | --- | --- |
| **Never** | 1.00 | 1.00 |
| **1-5** | 1.71 ± 0.11 | 1.87 ± 0.06 |
| **6-13** | 2.18 ± 0.13 | 2.39 ± 0.07 |
| **14-22** | 2.04 ± 0.13 | 2.22 ± 0.07 |
| **>22** | 1.82 ± 0.11 | 1.98 ± 0.06 |

‡ Adjusted for age (continuous) , menopause status (pre-/post-menopause), parity (0, 1, 2, ≥3 children), alcohol use(none, former, current), educational level (less than high school, high school, university), and BMI (quartiles).
